# ‘MATRI-SUMAN’ a capacity building and text messaging intervention to enhance maternal and child health service utilization among pregnant women from rural Nepal: study protocol for a cluster randomised controlled trial

**DOI:** 10.1186/s12913-018-3223-6

**Published:** 2018-06-14

**Authors:** Jitendra Kumar Singh, Rajendra Kadel, Dilaram Acharya, Daniel Lombard, Saval Khanal, Shri Prakash Singh

**Affiliations:** 1 Department of Community Medicine, Institute of Medical Sciences, Banaras Hindu University, Varanasi, Uttar Pradesh India; 2 Personal Social Services Research Unit, London School of Economics and Political Science, London, UK; 3 Department of Preventive Medicine, College of Medicine, Dongguk University, Seoul, Republic of Korea; 4 MedSolution Consultants, Sankalpa Foundation (Pvt) Ltd, Kaski, Nepal; 5 Department of Community Medicine and Public Health, Tribhuvan University, Janaki Medical College, Janakpur, Nepal; 6 School of Pharmacy, University of Queensland, Brisbane, Australia; 7 Department of Community Medicine, Kathmandu University, Devdaha Medical College and Research Institute, Devdaha Municipality, Rupandehi, Devdaha, Nepal

**Keywords:** FCHV, Capacity building, Text messaging, MCH service utilization, Nepal

## Abstract

**Background:**

Capacity development of health volunteers and text messaging to pregnant women through mobile phones have shown improved maternal and child health (MCH) outcomes and is associated with increased utilisation of MCH services. However, such interventions are uncommon in Nepal. We aim to carry out an intervention with the hypothesis that capacity building and text messaging intervention will increase the MCH service utilisation.

**Method/design:**

MATRI-SUMAN is a 12-month cluster randomized controlled trial (RCT). The trial involves pregnant women from 52 clusters of six village development committees (VDCs) covering 66,000 populations of Dhanusha district of Nepal. In the intervention clusters, Female Community Health Volunteers (FCHVs) will receive capacity development skills through reinforcement training, supervision and monitoring skills for the promotion of health seeking behaviour among pregnant women and study participants will receive periodic promotional text messaging service about MCH components through mobile phones. A sample of 354with equal numbers in each study arm is estimated using power calculation formula. The primary outcomes of this study are the rate of utilization of skilled birth attendants and consumption of a specified diversified meal. The secondary outcomes are: four antenatal (ANC) visits, weight gain of women during pregnancy, delivery of a baby at the health facility, postnatal care (PNC) visits, positive changes in child feeding practices among mothers, performance of FCHVs in MCH service utilization.

**Discussion:**

The intervention is designed to enhance the capacity of health volunteers for the promotion of health seeking behaviour among pregnant women and text messaging through a mobile phone to expecting mothers to increase MCH service utilization. The trial if proven effective will have policy implications in poor resource settings.

**Trial registration:**

ISRCTN60684155, (10.1186/ISRCTN60684155). The trial was registered retrospectively.

**Electronic supplementary material:**

The online version of this article (10.1186/s12913-018-3223-6) contains supplementary material, which is available to authorized users.

## Background

Maternal mortality is still the second largest causes of death among women:1 in every 180 women have chances of dying from maternal causes despite remarkable progress over the past decade [1]. Global communities have moved onto a new agenda of Sustainable Development Goals (SDGs) to ensure healthy lives and promote well-being for all at all ages and SDGs goal 3 targets to reduce the global maternal mortality ratio to less than 70 per 100,000 live births by 2030 [2, 3]. Despite a significant reduction of maternal death, Nepal needs greater effort to achieve the recommended reduction of maternal mortality ratio set by SDGs as the current maternal mortality ratio in Nepal is 170 per 100,000 live births [4].

Nepal Demographic and Health Survey 2011 revealed that only half of pregnant women in Nepal managed to attend four Antenatal Care (ANC) visits as recommended, about two-thirds of births (63%) took place at home, and only 45% of the mothers had postnatal visits [4]. Studies from Nepal and Sri Lanka showed that the anthropometric assessment of mothers and intake of micronutrients during pregnancy are associated with reduced maternal and childhood morbidity and mortality [5, 6]. The role of Female Community Health Volunteers (FCHVs) to promote safe motherhood, child health, family planning, and other community-based health services have been widely recognised in Nepal [7, 8]. These types of health volunteers can contribute to the useful bridge between the community and the primary health care facilities [9].

Studies from several developing countries have demonstrated that the use of trained health volunteers can have potential health benefits to mothers and children, such as increased uptake of routine immunization, breastfeeding, reduction in maternal and child morbidity and mortality, and increases in family planning services [10]. A study from Nepal found that the utilization of trained community health volunteers contributed to an improved skills to identify and manage a range of common diseases such as diarrhoea, night blindness, malnutrition and acute respiratory infections [11]. In a neighbouring country (India), trained community mobilizers were able to identify and counsel at risk families and there was significant improvement of breastfeeding techniques and duration, and the reduction of severe malnutrition cases among young children [11, 12]. Similarly, studies from African countries also concurred above findings [13, 14]. These evidences suggest that capacity building of health volunteers (especially FCHVs) along with some educational interventions for mothers might have significant benefits to the society in improving MCH services.

In recent years, mobile phones for information and communication in health sector has been increasingly used and became more popular in the developed as well as developing nations [15]. A global survey by WHO identified that “mHealth” initiatives are used in several countries in different forms and level of applications [16]. Evidence showed that mobile text messaging to educate people about healthy living practices can have a good impact on disease prevention and control, development of positive health behaviour, and better health service utilization [17–19]. For example, two Tanzanian studies [20, 21] demonstrated that the rate of ANC visits and utilisation of skilled health workers during delivery was improved with the implementation of text messaging services in resource-poor settings. Therefore, we aim to evaluate the impact of text messaging services coupled with capacity building to FCHVs in improving MCH services in rural areas of resource constraint district of Nepal.

The objective of this study is to evaluate the effectiveness of capacity building training and technological interventions (text messaging) for MCH service utilisation among rural women during antenatal and postnatal periods. Specific research questions consist of FCHVs’ performance on MCH service, service utilisation by pregnant women, balanced diet intake by pregnant women and breastfeeding practices. We hypothesize that capacity building of health volunteers and text messaging to pregnant women about MCH services help to improve the performance of FCHVs for the provision of MCH services, and service utilisation (e.g., antenatal and postnatal visits) and intake of healthy balanced diet by women during their antenatal and postnatal periods.

## Methods

### Study setting

The study is conducted across 6 VDCs (52 wards) of Dhanusha district of Nepal. Dhanusha is one of the 75 districts of Nepal, which is situated in the southern part (also known as ‘Terai’ in local language). VDCs are the basic political unit of the district, which is further divided into nine smaller units called wards. Each ward functions as one cluster in our study. The study area is predominantly inhabited by rural communities with agriculture as a main source of occupation (particularly vegetable farming) and the study population are relatively stable.

The district borders with Siraha, Mahottari and Sindhuli districts in the east, west and north, respectively and India in the south. The main residents of the district are from the Maithili heritage and the adult literacy rate is 69%. Administratively, the district comprises one municipality and 101 VDCs with an estimated population of 754,777 in 2011 [22]. Each VDC consists of one government health facility. According to the skilled birth attendant (SBA) policy of Nepal, the health post serves as the first contact point (birthing centre) for institutional delivery [4, 23], with the deployment of at least one staff nurse. Azonal hospital (200 beds) and a private medical college act as referral hospitals for Dhanusha and adjoining districts [24, 25].

### Study population

The study population consists of pregnant women aged between 15 and 49 years in their second trimester (gestation period between 13 and28 weeks). Pregnancy status was confirmed by a validated pregnancy test report. The gestational age will be determined based on first date of last menstrual period by asking with the participant. The expected date of delivery (EDD) based on gestational age will be confirmed through physical examination of pregnant women by nursing staff. Women were excluded if they had the following characteristics: (a) those not planning to stay at the study site during pregnancy and postpartum, (b) any physical and mental disability (which can adversely influence the study results), (c) women who have miscarriage or still birth during recruitment process, and (d) woman who are not willing (to give consent) to participate in the study.

### Study design

This is a two-parallel arms **cluster RCT** in rural community settings, provided participants are eligible for the study. Participants are recruited from 52 study areas by research assistants with the help of FCHVs. Then participants are then randomly assigned into intervention group and control arms based on clusters with approximately equal number of participations in each group.

**Fig. 1:**
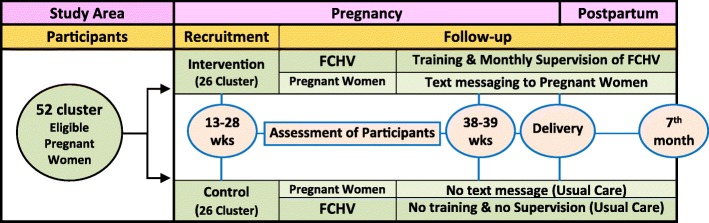
Trial Design

Baseline information is collected after allocation of the clusters in two groups. At the same time, one-day extensive reinforcement training to the FCHVs in the intervention arm is conducted. Intervention group will receive text messages in their mobile phones. The principal investigator is responsible for sending text messages to the participants once they are recruited in the study and this process will continue until the fifth-month after childbirth. There will be three assessments during follow-up period and the data will be collected during these assessment periods: between 38 and 39 weeks of gestation, during delivery, and at 7 months after child birth. During the follow-up period, monitoring and supervision of FCHVs in the intervention arm will also be done to assess their performance. Additionally, assessment of child will be carried out at birth and 7 months after birth [Fig. 1].

### Sample size estimation

The estimation of sample size was done considering the study design as two-parallel arm cluster RCT [26]. We assumed that 12% of the child births have been attended by a skilled birth attendant (SBA) at baseline in both arms and this rate will increase to 32% post- intervention in the intervention arm. 20% as the minimum difference between the two groups we would like to see after intervention with 0.80 power and 0.05 level of significance, and to account for intra-cluster correlation by taking the design effect of 2.29 for delivery attended by SBA, an adjusted sample size of 147 pregnant women in each arm is required for the study [4, 24]. With allowable 20% potential attrition, the final sample size is 177 for each arm. Thus, 354 pregnant women are estimated to be included in a trial. This number would be sufficient to detect a change in the effect sizes over a follow-up period in the intervention group as compared to control group. The study area consisted of 67,625populations (male/female ratio = 100:94) and the reported birth rate are 25.5 per 1000 per year [4], which gives an estimated 1724 expected pregnant women. Since, this study will consider the pregnant women in their second trimester, around 431 (=1724 × 0. 25) pregnant woman would be available at any point of time during a year, which is sufficient number for our study.

### Sampling technique

We employed multi-stage cluster sampling. First, two health facilities (consisting of one primary health care centre and one health post) catchment area of Dhanusha district has been selected purposefully. We selected these health facilities because there have been no MCH-related services from other organizations before, but the governmental. Furthermore, no research work has been conducted yet relating to this field.

Second, six VDCs have been selected in the catchment area of these two health care facilities by stratified random sampling technique. The population of each VDC in the catchment area range from 3500 to 19,000. Therefore, VDCs in both health care facilities catchment areas have been stratified into three strata based on population size (< 5000, 5000–10,000, and > 10,000), forming a total of six strata. One VDC has been randomly selected from each stratum [24, 25]. Third, those selected six VDCs comprised of 54 wards (here we called cluster for our study) where two of them are excluded as these wards are situated in the town area. The reminder 52 clusters have been selected for a trial. Finally, the complete enumeration of all households with pregnant women in their 2nd trimester from these clusters has been carried out.

### Identification and recruitment of participants

All households with pregnant women in 52 clusters have been identified and screened applying inclusion and exclusion criteria. The identification of pregnant women has been made with the help of FCHVs’ antenatal registers. Pregnant women from each household in have been registered by FCHVs in their respective ward. All pregnant women with gestational period between 13 to 28 weeks are included in the study. Each household is coded and marked in the outside wall with a visible unique serial number to track participants during follow-up period.

The baseline information of these pregnant women was collected between July and September 2015. Selection of participants has been done by trained female research assistants (FRA), who are fluent in both Nepali and Maithili languages. Written informed consent have been taken from each participant who are willing to participate in the study. Participants are informed about the purpose of the study, advantages and any potential harms arising from a study. Head of the family, usually husband or close relatives of the relevant participants, were also informed about the study. For any adverse event or inquiry by the participants will be entertained or clarified.

### Matching

Before baseline data collection, pre-trial inventory on antenatal, delivery and postnatal care, and number of pregnancies in the study area have been obtained from respective health facilities and FCHVs’ records. We have formed matched pair of clusters consulting with village secretary, chairman of the ward and FCHV of the respective ward. There are three criteria for matching: approximately equal numbers of pregnant women in the cluster; similar or predominant ethnic group distribution in each cluster; and proportionate SBA utilization rate across the cluster. The matched pair of cluster is made within each VDC. As there are nine wards in each VDC, an odd number of clusters in the VDC have been matched with odd number of cluster with other VDC. We have selected 26 matched pairs from52 clusters of the study area. Finally, 26 pairs of clusters were randomized into intervention and control arms, each arm holding 26 clusters.

### Randomization

Twenty-six pairs of matched clusters have been ranked from the top best matched to the bottom matched based on matching criteria. Within each pair, the random allocation rule has been used to assign either to the intervention group or control group (Additional file 1). There is no blinding of the researchers and participants in the study due to the nature of the intervention and ethical reasons. Data collectors during follow-up will be blinded to allocation.

### Potential contamination

Most of the clusters are geographically separated and that the distance between clusters are at least 500 m from each other which may prevent the possible treatment contamination between intervention and control arms. There may be potential contamination due to sharing of training knowledge but knowledge is not equal to implementation, moreover, the control group FCHVs will not be followed-up by our FRAs. The likely contamination of text messaging through mobile phone to the pregnant women will be confirmed by asking whether they received MCH information from the intervention area during follow-up period.

### Interventions

The intervention has been implemented by two approaches; i) Capacity building of FCHVs (reinforcement training on maternal and new-born health followed by regular supervision) for the promotion of health seeking behaviour among pregnant women, and ii) periodic text messaging to the pregnant women through a mobile phone for demand generation of MCH services.

#### Capacity building of FCHVs

##### Reinforcement training

FCHVs have received one-day extensive orientation/reinforcement training to equip with the knowledge and skills of MCH services. The content of the reinforcement training to FCHVs comprised of three packages; maternal and neonatal health (MNH) counselling package “Jeevan Suraksha” also called “birth preparedness package” developed by USAID; nutrition training package developed by ministry of health/micronutrient initiative, Nepal [27]; and “Bal Parivar Mitra” also called “baby friendly family”, a module for community-based maternal and child health nutrition (CB-MCHN) project [28].We provided daily allowance and refreshment to FCHVs during training course. FCHVs who have attended the training and actively involved throughout training day. We have conducted pre- & post-test of the trainees (FCHVs) and found good improvement in their knowledge regarding training course.

##### Supervision and monitoring

A monthly visit to FCHVs in the intervention areas will be made by researchers using supervision and monitoring format adopted from “Bal Parivar Mitra” monitoring format for the CB-MCHN project (motivation for change) in India [28]. The FCHVs will be assessed by researchers against their performance on MCH services delivery (Additional file 2).

#### Text messaging through a mobile phone

A short text message will be sent regularly through a mobile short messaging service (SMS) system to either pregnant woman or her family member (who can convey messages to the participant) in the intervention arm. The start point of sending text message is on the individual basis and the process starts immediately after participants got recruited and allocated in the study. Participants in this arm will be provided with a diary and a pencil and will be advised to note down messages received by them. Text messages will be either in Nepali or in Maithili version based on participant’s language preference. The frequency will be one message every fortnight between 4th and 6th months and every week thereafter till delivery of the baby. The enquiry will be made within a week after the expected date of delivery (EDD) for the outcome of pregnancy by research assistants. One message per week will be sent during the postnatal period (up to five months after delivery). The content of SMS is about dates of ANC / PNC visit, care during pregnancy, danger signs in pregnancy, postnatal care and care of new born baby including breastfeeding.

A package of common messages of 160 or fewer letters has been prepared for all participants in the intervention arm according to the stage of pregnancy and the postnatal period, focussing on routine antenatal care, place of delivery, postnatal care and diversified dietary intake including how frequently they consume such diet. All together 5 ‘message packages’ were created and each package contained 5 relevant information (Additional file 3). All the messages have been created according to stage of the pregnancy and postpartum period adopted from the training materials for “Jeevan Suraksha” (Additional file 4), Micronutrient Initiative-Nepal and motivation for change from CB-MCHN project [27, 28]. The timing of text messaging will be either 6:00–9:00 AM or 6.00–9:00 PM as participants are generally free during this period.

Both the intervention and control groups will receive regular antenatal and postnatal care provided by the government through local health facilities. These services are free of cost at the point of delivery. Both intervention and control arms participants should make at least four ANC visits (first within 4 months, second within 6 months, third within 8 months and fourth within 9 months, respectively in nearby health facility) and three home visits (First visit at day one, second at day 3 and third at day 7) in the first 7 days after childbirth by FCHVs/CHWs as part of postnatal care service.

**Usual care:** FCHVs in the control clusters will not receive training on the MCH service package and participants (pregnant women) also will not receive any text message during study period. There will be no supervision to the FCHVs from the project side, but the monthly visit by MCHWs/CHWs to them has been made from the government side as part of regular supervision to collect integrated community health service reports including MCH service reports. Any adverse event.

### Outcome measures

#### Primary and secondary outcomes of the study include;

Primary Outcomes

1. Utilization of Skilled Birth Attendant (SBA) by pregnant women during delivery.
2. Consumption of diversified diet by women during pregnancy and postpartum.

Secondary Outcomes

1. Four ANC visits by pregnant woman
2. Delivery of baby in a birthing centre (health care facility)
3. PNC visits by mother and child
4. Weight gain, haemoglobin changes and increase in uterine height of woman during pregnancy
5. Changes in child feeding practices among mothers, and
6. Performance of FCHVs

Details of outcome measures are given in Additional file 5.

### Research instruments

Structured and semi-structured questionnaires were adapted from NDHS 2011 to collect quantitative information [4]. There are three sections in a questionnaire: i) baseline questionnaire ii) antenatal follow-up questionnaire, and iii) postnatal follow-up questionnaire. The baseline questionnaire includes three components: i) socioeconomic information, ii) obstetric/gynaecologic history, anthropometric measurement and existing service utilization of pregnant women, and iii) Food Frequency Questionnaire (FFQ) and dietary diversity questionnaire recommended by the UN Food and Agriculture Organisation [29]. The antenatal follow-up questionnaire also consists of three components: i) information on services utilization, ii) anthropometric measurement and iii) FFQ and dietary diversity questionnaire. Postnatal follow-up questionnaire consists of two components i) maternal and neonatal services utilization along with morbidity and mortality and ii) new-born anthropometric measurement and child care practices. A focus group discussion (FGD) guide is prepared to collect information on attitudes and beliefs of FCHVs and pregnant women on service utilization and nutrition practices to give an in-depth understanding on the status of dietary diversity and complement some of the quantitative findings.

### Anthropometric measurement

Anthropometric variables: All the anthropometric measurements will be taken on each pregnant women and their subsequent child using standard anthropometric measurement techniques [30].

### Haemoglobin estimation

A total of 2 ml venous blood will be collected and kept in EethyleneDiamine Tetra-acetic Acid (EDTA) vial. Collected EDTA blood will be transferred to the clinical pathology laboratory of Janaki Health Care and Research Centre, Janakpur and Haemoglobin concentration will be estimated by Cyanmethemoglobin method.

### Data collection plan

The structured questionnaire has been piloted using responses from 20 households in the adjacent district. Necessary feedback is received and the amendment has been made in the final questionnaire before baseline data collection. Data will be collected by research assistants who are trained in the tools and techniques of data collection. Anthropometric assessment will be taken by six Female Research Assistants (FRAs) (one FRA per VDC).All measurements will be made three times and the median value will be recorded for analysis. The collection of information on pregnancy, outcomes and its determinants will be made longitudinally at different stages. The methods will employ structured interviews, FGD and anthropometric assessment.

Data will be collected at four chronological points. Baseline information on demographic characteristics, obstetric history, anthropometry, biochemical test, dietary survey and current practices on services utilization will be taken on 4–6 month prior to intervention. The reminders will be the follow-up period as described below;

1. Assessment of pregnant women (services utilization, dietary survey, anthropometry and biochemical test) in the 9th month during intervention (Follow up- I).
2. Data on birth weight will be taken by FRAs preferably within the first hour of the birth (Follow up II).
3. Assessment of mother and child (services utilization, dietary survey and anthropometry including maternal and child morbidity) and satisfaction of the participants at the7th month after intervention (Follow up III).

### Data management and analysis plan

Data entry will be performed in EpiData software version 3.1 and then it will be transferred to STATA software (Version 13.0) where all statistical analyses will be performed. The logistic regression model will be used to assess primary and secondary outcomes of the intervention. The independent t-test will be used to compare mean of different variables between the intervention and control groups. Chi-square test will be used to investigate the association between categorical variables in intervention and control groups. Within group analysis will also be done for outcomes whether it is attributed to either text message or capacity building or both.

For the repeated anthropometric measurement, biochemical test and dietary measures, we will use generalized estimating equation (GEE) and mixed model analysis to evaluate the effects of the intervention. The analysis will adjust for potential confounders for changes in weight gain and haemoglobin including baseline socio-demographic characteristics. We will also evaluate the possible interactions between the intervention indicators and participant attributes to assess the effect of intervention. Data will be analysed by ‘intention-to-treat’ principle.

Multiple imputations will be performed to account for missing data. Results will be expressed as odds ratios (ORs) with 95% confidence intervals for primary and secondary binary outcomes. Exploratory analysis will also be carried out on participant’s sub-groups to address heterogeneity. All test will be two-tailed and *p*-value will be set at < 0.05 for statistical significance.

All the FGDs will be recorded in a voice recorder. A coding framework will be prepared based on FGDs guideline. Transcription of data will be done in Nepali (National language), and then will be translated to English. Two researchers will be involved separately for coding based on pre-identified theme in the evaluation framework. Coding will be compared for consistency. The analysis will be done manually and no other software will be used for qualitative data analysis.

### Quality control

To ensure the quality, this study will use standard operating procedures for major processes. The field study team will receive extensive training on the objectives, methods and procedures as well as ethics coupled with frequent supervision and monitoring.

## Discussions

Maternal and child health programme is one of the priority programmes of government of Nepal as this programme is included in the SDGs. Government in developing countries, including Nepal, with significant assistance from the international bilateral and non-government organisations, have been attempting to ensure the maternal and child health care services through various plans and programmes. Efficacy of safe motherhood programme is exacerbated due to several hindrances in practical aspects at grass root level. There is still a huge gap in the provision of equity-based maternal and child health services in rural Nepal. The maternal and child health interventions for people with lower socioeconomic status residing in rural areas should be identified to recommend coherent policy alternatives.

Therefore, this trial is initiated with the main objective to assess the effectiveness of capacity building and text messaging intervention on MCH service utilization and improvement in dietary intake among pregnant women in rural communities of Dhanusha district of Nepal and it is expected that this intervention will enhance the service utilization and dietary intake among intervention group as compared to control. The unique feature of this trial is that we are focusing on two components of the intervention simultaneously. First, the capacity building of FCHVs will help to support pregnant women to enforce and promote health seeking behaviour. Second, at the same time, text messaging through mobile phone will generate demand and create awareness for service utilization and optimal intake of balanced and diversified diets among pregnant women in the study area.

This trail is named as MATRI-SUMAN (Maternal Alliance for Technological Research Initiative on Service Utilization and Maternal Nutrition) with two Sanskrit words; one belongs to mother and another to the child. Survival and well-being of both the mother and newborn child effective depend upon maternal and child health care. Information on available services, their utilization and modification of dietary intake by pregnant and postpartum women may help to reduce mortality and morbidity in both mother and child. Identification of pregnant women, particularly underprivileged and have inadequate access to health care services, are needed to increase the effectiveness of preventive programmes. The impact of such intervention is likely to be the best alternatives in women in resource limited settings such as rural part of Nepal. This trial uses an eminent access, low cost strategy, and easily acceptable technologies which can be scalable into wider community settings in underprivileged population. This protocol will serve as guidance for future researchers to develop effective strategies to study on maternal and child health related services in rural Nepal and other resource limited settings.

### Trial status

This trial MATRI-SUMAN is designed for the PhD research Project in 2014–17 and is currently completed.

## Additional files

Additional file 1: **Table S1.** Distribution of 52 clusters in Intervention and Control arm (DOCX 30 kb)

Additional file 2: Formats- Female Community Health Volunteers (FCHV) monitoring formats. (PDF 2834 kb)

Additional file 3: **Table S2.** Package of Messages for Various Stage of Pregnancy and Postpartum. (DOCX 31 kb)

Additional file 4: Documents - from which text messages are prepared. (PDF 823 kb)

Additional file 5: **Table S3.** Indicators for outcome measurement in intervention and control group. (DOCX 31 kb)

Additional file 6: Participant Consent Form. (DOCX 15 kb)

## Abbreviations

ANC: Antenatal Care
CB-MCHN: Community Based- Maternal and Child Health Nutrition
EDD: Expected Date of Delivery
FCHV: Female Community Health Volunteer
FFQ: Food frequency questionnaire
FGD: Focus Group Discussion
FRA: Female Research Assistant
IDI: MHN Maternal and Neonatal Health
MATRI-SUMAN: Maternal Alliance for Technological Research Initiative on Service Utilization and Maternal Nutrition
MCH: Maternal and Child Health
MDG: Millennium Development Goal
NDHS: Nepal Demographic and Health Survey
NHRC: Nepal Health research Council
PHCC: Primary Health Care Centres
PNC: Postnatal Care
SBA: Skilled Birth Attendant
SMS: Short Messaging Services
UN: United Nations
VDC: Village Development Committee

## References

[1] Boerma T, Mathers C, AbouZahr C, Chatterji S, Hogan D, Stevens G, Mahanani W, Ho J, Rusciano F, Humphreys G: Health in 2015: from MDGs millennium development goals to SDGs sustainable development goals. 2015.

[2] Mahato PK, Van Teijlingen E, Simkhada P, Angell C (2016). Birthing centres in Nepal: recent developments, obstacles and opportunities. Journal of Asian Midwives.

[3] Nations. U (2015). Transforming our world: The 2030 agenda for Sustain Dev In.

[4] Ministry of Health and Population, New ERA, ICF International (2012). Nepal Demographic and Health Survey 2011 Kathmandu, Nepal and Calverton, Maryland.

[5] Jananthan R, Wijesinghe D, Sivananthawerl T (2009). Maternal anthropometry as a predictor of birth weight. Trop Agric Res.

[6] Christian P, Stewart CP, LeClerq SC, Wu L, Katz J, West KP, Khatry SK (2009). Antenatal and postnatal iron supplementation and childhood mortality in rural Nepal: a prospective follow-up in a randomized, controlled community trial. Am J Epidemiol.

[7] Glenton C, Scheel IB, Pradhan S, Lewin S, Hodgins S, Shrestha V (2010). The female community health volunteer programme in Nepal: decision makers’ perceptions of volunteerism, payment and other incentives. Soc Sci Med.

[8] Department of Health Service, Ministry of Health and Population: Annual Report, 2013/2014. In: *Annual Report, 2013/2014,.* Kathmandu, Nepal: Department of Health Services & Ministry of Health and Population; 2013–2014.

[9] Maru RM (1983). The community health volunteer scheme in India: an evaluation. Soc Sci Med.

[10] Lewin S, Babigumira S, Bosch-Capblanch X, Aja G, Van Wyk B, Glenton C, Scheel I, Zwarenstein M, Daniels K (2006). Lay health workers in primary and community health care: a systematic review of trials.

[11] Curtale F, Siwakoti B, Lagrosa C, LaRaja M, Guerra R (1995). Improving skills and utilization of community health volunteers in Nepal. Soc Sci Med.

[12] Gisore P, Shipala E, Otieno K, Rono B, Marete I, Tenge C, Mabeya H, Bucher S, Moore J, Liechty E (2012). Community based weighing of newborns and use of mobile phones by village elders in rural settings in Kenya: a decentralised approach to health care provision. BMC pregnancy and childbirth.

[13] Brenner JL, Kabakyenga J, Kyomuhangi T, Wotton KA, Pim C, Ntaro M, Bagenda FN, Gad NR, Godel J, Kayizzi J (2011). Can volunteer community health workers decrease child morbidity and mortality in southwestern Uganda? An impact evaluation. PLoS One.

[14] Mushi D, Mpembeni R, Jahn A (2010). Effectiveness of community based safe motherhood promoters in improving the utilization of obstetric care. The case of Mtwara Rural District in Tanzania. BMC pregnancy and childbirth.

[15] Kahn JG, Yang JS, Kahn JS (2010). ‘Mobile’health needs and opportunities in developing countries. Health Aff.

[16] Kay M, Santos J, Takane M. mHealth: new horizons for health through mobile technologies. World Health Organization. 2011:66–71.

[17] Kaplan WA (2006). Can the ubiquitous power of mobile phones be used to improve health outcomes in developing countries. Glob Health.

[18] Free C, Phillips G, Galli L, Watson L, Felix L, Edwards P, Patel V, Haines A (2013). The effectiveness of mobile-health technology-based health behaviour change or disease management interventions for health care consumers: a systematic review. PLoS Med.

[19] Gurol-Urganci I, de Jongh T, Vodopivec-Jamsek V, Atun R, Car J. Mobile phone messaging reminders for attendance at healthcare appointments. Cochrane Libr. 2013; 10.1002/14651858.CD007458.pub3 PMC6485985 24310741

[20] Lund S, Hemed M, Nielsen BB, Said A, Said K, Makungu M, Rasch V (2012). Mobile phones as a health communication tool to improve skilled attendance at delivery in Zanzibar: a cluster-randomised controlled trial. BJOG Int J Obstet Gynaecol.

[21] Lund S, Nielsen BB, Hemed M, Boas IM, Said A, Said K, Makungu MH, Rasch V (2014). Mobile phones improve antenatal care attendance in Zanzibar: a cluster randomized controlled trial. BMC pregnancy and childbirth.

[22] CBS: General and Social Characteristics Tables (2014). Household and Population, Age-Sex Distribution, Relationship, Marital Status and Religion,National Population and Housing Census 2011.

[23] MOHP (1991). National Health Policy, Ministry of Health and Population(MOHP).

[24] District Health Profile of Dhanusha, Nepal. In*.* Dhanusha, Nepal: District Health Office, Dhanusha, Ministry of Health and Population; 2012/2013.

[25] District Development Committee Dhanusha, Ministry of Local Development: A profile of Dhanusha District of Nepal. In*.* Janakpur, Nepal: Ministry of Local Development, & District Development Committee, Dhanusha; 2012: 1–4.

[26] Chan Y (2003). Randomised controlled trials (RCTs)-sample size: the magic number?. Singap Med J.

[27] McPherson RA, Tamang J, Hodgins S, Pathak LR, Silwal RC, Baqui AH, Winch PJ (2010). Process evaluation of a community-based intervention promoting multiple maternal and neonatal care practices in rural Nepal. BMC pregnancy and childbirth.

[28] Community Based Maternal & Child Health Nutrition (MCHN) Project Evaluation Report. In*.* Lucknow: ORG Centre for Social Research (A Division of ACNielsen ORG-MARG Private Limited); 2006.

[29] FAO. DIETARY DIVERSITY QUESTIONNAIRE: In*.*: FAO/Nutrition and Consumer Protection Division; 2007.

[30] Weiner JS, Lourie JA (1981). Practical human biology: academic Pr.

