# Supplementary material for: ‘MATRI-SUMAN’ a capacity building and text messaging intervention to enhance maternal and child health service utilization among pregnant women from rural Nepal: study protocol for a cluster randomised controlled trial
Source: BMC Health Serv Res. 2018 Jun 14;18:447. doi: 10.1186/s12913-018-3223-6 (PMC6001039; doi:10.1186/s12913-018-3223-6)
Supplement: Supplementary file 1 — Table S1. Distribution of 52 clusters in Intervention and Control arm (DOCX 30 kb) [file 12913_2018_3223_MOESM1_ESM.docx]

**Table 1: Distribution of 52 clusters in Intervention and Control arm**

| **Intervention arm** | **Control arm** |
| --- | --- |
| **Dhalkewar** | |
| Eklebar, Kemalipakha, Kemalipur, Hadeghari | Anandpur, Bhaktipur, Nayatole, Shivnagar |
| **Digambarpur** | |
| Auraha, Digambarpur, Nayatol, Sitanagar | Echhapur, Pathara, Naya Bazar, Ashoktol, |
| **Hariharpur** | |
| Bintol, Hariharpur, Labatoli, Magartol | Chhaghariatol,Durgatol, umalikhadi,Lalbhitti |
| **Sakhuwa** | |
| Arjanama, Hasanpur, Kumharaul, Parsa, | Chandrapur, Haraiya,Mangalpur, Pakadiya |
| **Shantipur** | |
| Akhilanandtol, Bintol, Durgasingtol, Jibastol. | Chandrapur, Dharatol, Kalipur, Kashatol |
| **Tulsi** | |
| Bahunmara,Bhandaritol,Jiraidanda, Bismargaun | Bimire, Dhadkholagaun, Lamidanda, Lotagaun. |
| **Digmabarpur, Hariharpur / Shantipur, Tulsi** | |
| Badadhuria, Pahaditol | Birpur, Hatigauda |
