# Supplementary material for: ‘MATRI-SUMAN’ a capacity building and text messaging intervention to enhance maternal and child health service utilization among pregnant women from rural Nepal: study protocol for a cluster randomised controlled trial
Source: BMC Health Serv Res. 2018 Jun 14;18:447. doi: 10.1186/s12913-018-3223-6 (PMC6001039; doi:10.1186/s12913-018-3223-6)
Supplement: Supplementary file 2 — Formats- Female Community Health Volunteers (FCHV) monitoring formats. (PDF 2834 kb) [file 12913_2018_3223_MOESM2_ESM.pdf]

Formats: Female Community Health Volunteers (FCHV) monitoring formats

Format-1: FCHV Monitoring Format (During Pregnancy)

	**Number of Pregnant women Identified =..............**	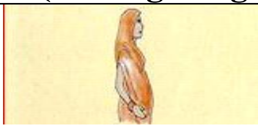	**Number of Pregnancy Registered=..................**
	No. of Pregnant Women who got suggestions from FCHV	***Suggestions***	***No. of Pregnant Women who following the Suggestions of FCHV***
	Suggestion For: 4 ANC ◯	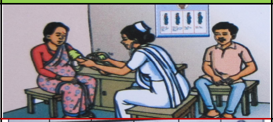	*No. of Pregnant Women receiving ANC* ☐
	Suggestion For: IFA ◯	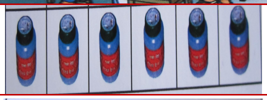	*No. of Pregnant Women taking IFA Tablets* ☐
	Suggestion For: TD ◯	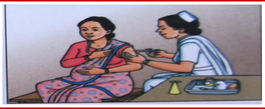	*No. of Pregnant Women taken TD* ☐
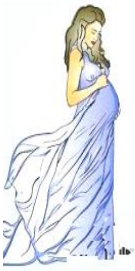	*Suggestion For: De-worm* ◯	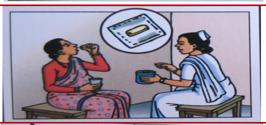	*No. of Pregnant Women taken De-worming tablets* ☐
	Suggestion For: Additional & nutritious diet ◯	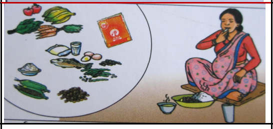	*No. of Pregnant Women taking additional and nutritious diet* ☐
**Suggestions for Pregnant Women**	*Suggestion For: No Alcohol & Smoking* (Among Alcoholism & Smokers) ◯	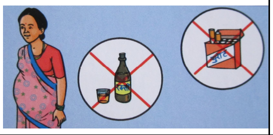	*No. of Pregnant Women who quit Alcohol & Smoking* ☐
	Suggestion For: Rest & Sleep after Lunch ◯	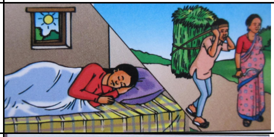	*No. of pregnant Women taking Rest and Sleep after Lunch* ☐
	Suggestion For: Personal Hygiene. ◯	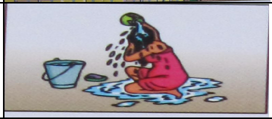	*No. of pregnant Women practicing personal hygie* ☐
	Suggestion For: Safe Delivery ◯	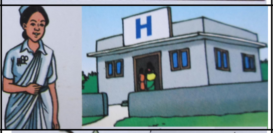	*No. of pregnant Women having Safe Delivery* ☐
	Suggestion For: Prepared with Money & Transportation ◯	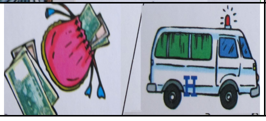	*No. of pregnant Women Prepared with Money & Transportation* ☐

Format-2:FCHV Monitoring Format (During Post-natal period)

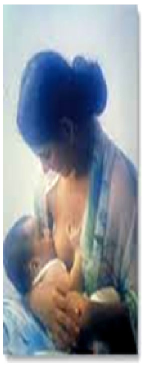 **Suggestion for Postnatal Mother & Newborn babies**	***No. of Post-natal Women who got suggestions from FCHV***	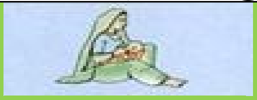	***No. of Post-natal Women who following the Suggestions of FCHV***
	Suggestions for New Born Babies:		
	Suggestion: To Clean with dry /soft cloths & to Keep baby warm ◯	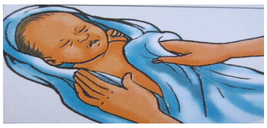	No. of Newborn cleaned with Soft & Dry cloths and kept warm. ☐
	Suggestion: To Put the baby to the mother chest. ◯	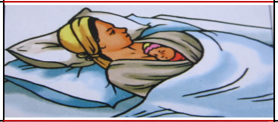	No. of Newborn put to their mothers Chest. ☐
	Suggestion: Not to bath the baby for 24 hour ◯	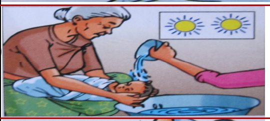	No. of Newborn having no bath within 24 hour of birth. ☐
	Suggestion: Not to apply anything other to the chord of baby ◯	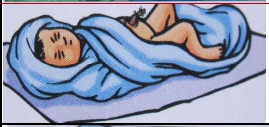	No. of Newborn with no application of anything ot to their chord. ☐
	Suggestion: To Initiate breast milk within one hour & to fe colostrums. ◯	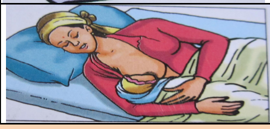	No. of Newborn initiated breast milk within one hour & fed colostrums. ☐
	Suggestions for Post Natal Mothers:		
	Suggestion For: 3 PNC (Mother & Newborn) ◯	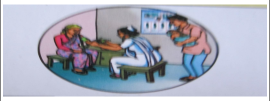	No. of Mothers & babies visited 3 PNC ☐
	Suggestion For: IFA tablet ◯	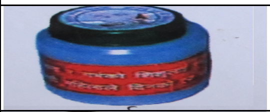	No. of Mothers took IFA Tablets. ☐
	Suggestion For: Vitamin A capsule ◯	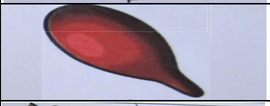	No. of Mothers took Vitamin A capsule. ☐
	Suggestion For: Additional & Nutritious Food ◯	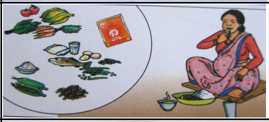	No. of Mothers took Additional & Nutritious Food ☐
	Suggestion For: Exclusive Breastfeeding ◯	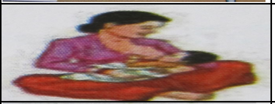	No. of Mothers practicing Exclusive Breastfeeding. ☐
	Suggestion For: Complementary Feeding ◯	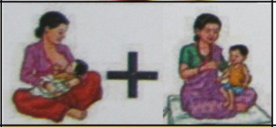	No. of Mothers who has given Complementary feeding in time ☐
	Suggestion For: Immunization ◯	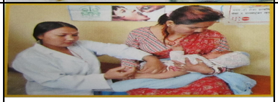	No. of Mothers who immunized their child. ☐
	Suggestion For: Growth Monitoring ◯	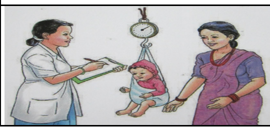	No. of Mothers who monitored growth for their baby. ☐
