# Supplementary material for: ‘MATRI-SUMAN’ a capacity building and text messaging intervention to enhance maternal and child health service utilization among pregnant women from rural Nepal: study protocol for a cluster randomised controlled trial
Source: BMC Health Serv Res. 2018 Jun 14;18:447. doi: 10.1186/s12913-018-3223-6 (PMC6001039; doi:10.1186/s12913-018-3223-6)
Supplement: Supplementary file 3 — Table S2. Package of Messages for Various Stage of Pregnancy and Postpartum. (DOCX 31 kb) [file 12913_2018_3223_MOESM3_ESM.docx]

**Table-2: Package of Messages for Various Stage of Pregnancy and Postpartum.**

|  | **Just after Recruitment** | **34^th^ week** | **37^th^ week** | **After delivery** |
| --- | --- | --- | --- | --- |
|  | ***Package-1*** | ***Package-2*** | ***Package-3*** | ***Package-4*** |
| **Service utilization** | Pregnant women should register herself at health facility or at qualified health professionals for health checkup. | Delivery should be conducted at Health Facility with help of SBA. | Delivery should be conducted at Health Facility with help of SBA. | Postpartum women should make at least 3 Visit for their (maternal & child) health checkup. First: within 24 hour, Second: 3^rd^ day & Third: 7 days. |
|  | Pregnant women should make at least 4 Visit for their health checkup. | As previous | New born should be wrapped with clean & soft cloth just after birth & do not put any- thing to chord except ointment. | Continue IFA for 45 days and take one dose of vit. A from health facility. |
|  | Pregnant women should take one IFA Tablet daily from 4^th^ month of pregnancy till 45 days of delivery (total 225 tablet) | As previous | Do not bath new for 24 hour. | Give exclusive breast feeding for 6 month |
|  | Pregnant women should take 2 dose of TD. | As previous | Initiate breast milk to your baby within one hour of birth | Give complementary feeding after 6 month along with breast milk. |
|  | Pregnant women should take one dose of De-worm after 4^th^ month of Pregnancy. | As previous | Do not give pre-lacteal feeding (like honey, grape water etc). | Immunize your child in time (all vaccines are available free at immunization centre). |
|  | ***Package-5*** |  |  |  |
| **Dietary intake** | Pregnant women should take one additional and diversified diet (from seven food group). [eg: cereals, pulses, vegetable, green leafy vegetables, fruits, milk & milk product, and egg/fish/meat or spourted legumes]. | As previous | As previous | As previous |
|  | It is better to take a piece of lemon or citrus fruit in your diet. | As previous | As previous | As previous |
|  | Do not take alcohol and smoking and Unnecessary drug. | As previous | As previous | As previous |
|  | Avoid hard work especially lifting heavy materials. | As previous | As previous | As previous |
|  | Take two hour rest after meal at daytime. | As previous | As previous | - |
