# Supplementary material for: ‘MATRI-SUMAN’ a capacity building and text messaging intervention to enhance maternal and child health service utilization among pregnant women from rural Nepal: study protocol for a cluster randomised controlled trial
Source: BMC Health Serv Res. 2018 Jun 14;18:447. doi: 10.1186/s12913-018-3223-6 (PMC6001039; doi:10.1186/s12913-018-3223-6)
Supplement: Supplementary file 4 — Documents - from which text messages are prepared. (PDF 823 kb) [file 12913_2018_3223_MOESM4_ESM.pdf]

Documents: from which text messages are prepared.

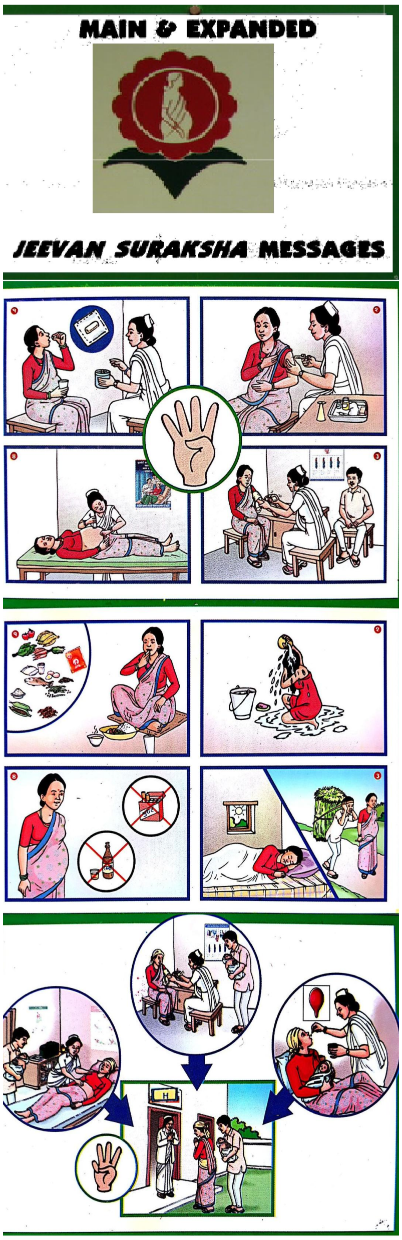

❷ **ANTENATAL CHECK-UP ACTION CARD**

MAIN MESSAGE

A pregnant woman should have at least 4 ANC visits:

1ST TIME : As soon as she knows she is pregnant
- Have her blood pressure checked
- Have an abdominal examination
- Have her first dose of TT

2ND TIME : Between the fifth to seventh month
- Have her blood pressure checked
- Have an abdominal examination
- Receive iron folate capsules, which should be taken every day from the fourth month of pregnancy until six weeks after delivery; and
- Have her second dose of TT

3RD TIME : During the ninth month
- Have her blood pressure checked
- Have an abdominal examination
- Receive iron folate capsules, which should be taken every day from the fourth month of pregnancy until six weeks after delivery

4TH TIME : Last month or during the week of delivery
- Have her blood pressure checked
- Have an abdominal examination
- Receive iron folate capsules, which should be taken every day from the fourth month of pregnancy until six weeks after delivery

A woman should have an antenatal examination anytime, whenever she feels or sees any problems.

MAIN MESSAGE

Immediately after the baby is born:

- She/he should be cleaned and wrapped in a cloth to keep warm.
- Breast- feeding should be started within an hour of delivery, after cleaning the breast of the mother.
- Nutritious food, emotional support and affection should be given to the mother by the family.
- The postnatal mother needs additional rest.

MAIN MESSAGE

In the first 24 hours after delivery, both the mother and baby should have a check-up at home, by a skilled healthcare provider. This should include:

- Uterus (abdominal) examination of the mother
- Respiratory functions check up of the newborn

After 7 days, both the mother and baby should have a check-up by a skilled healthcare provider:

- Uterus (abdominal) examination of the mother
- Temperature check
- Respiratory functions of the newborn
- BCG immunisation for the newborn

On the 42nd day after delivery, both the mother and baby should have a check-up by a skilled healthcare provider.
