# Supplementary material for: ‘MATRI-SUMAN’ a capacity building and text messaging intervention to enhance maternal and child health service utilization among pregnant women from rural Nepal: study protocol for a cluster randomised controlled trial
Source: BMC Health Serv Res. 2018 Jun 14;18:447. doi: 10.1186/s12913-018-3223-6 (PMC6001039; doi:10.1186/s12913-018-3223-6)
Supplement: Supplementary file 5 — Table S3. Indicators for outcome measurement in intervention and control group. (DOCX 31 kb) [file 12913_2018_3223_MOESM5_ESM.docx]

| **Outcomes** | | **Indicators (Intervention vs Control)** |
| --- | --- | --- |
| ***Primary Outcome*** | | |
| Utilization of Skilled Birth Attendant (SBA) by pregnant women at delivery. | | The % of births in which women delivered with a skilled birth attendant (doctor, ANM, or nurse). |
| Consumption of diversified diet by women during pregnancy and postpartum. | | % of women who consumed food from more than 7 food group according to FAO. |
| ***Secondary Outcomes*** | | |
| Four ANC visits by pregnant women | % of women who received 4 ANC visits from a skilled provider & other components which include:  % of women who took 225 IFA tablets.  % of women took de-worming,  % of women who immunized with 2 doses of TD.  % of women for whom Blood, Sool and Urine sample tested, and  % of women for whom anthropometric measurement taken. (Weight, BP) | |
| Delivery of baby in a birthing centre (health care facility) | The % of births that took place at health facility (public or private). | |
| PNC Visit by mother and child | % of births for which mother and new born received 1/2/3 postnatal check-up from skilled provider. | |
| Changes in haemoglobin level | % of pregnant women with haemoglobin concentration during pregnancy per g/dl per month. | |
| Weight gain of women during Pregnancy: | % of pregnant women with weight gain during pregnancy per kg / month (from the 4th month of pregnancy). | |
| Increase of uterine height | % of pregnant women with uterine height gain during pregnancy per cm/ week (from the 4th month of pregnancy). | |
| Changes in child feeding practices among women | % of women who initiate breast milk within ½ hour  % of women who fed colostrums to their baby.  % of women who did not give prelactal feedings.  % of women who practices exclusive feeding  % of women who started complementary feeding at 6 months. | |
| Performance of FCHV | % of FCHV given response on correct answers on MCH services | |
|  | % of FCHV reporting Regularly | |
|  | % of FCHV reporting Timely | |
|  | % of FCHV reporting Correctly | |

**Table 3: Indicators for outcome measurement in intervention and control group.**
