# Supplementary material for: ‘MATRI-SUMAN’ a capacity building and text messaging intervention to enhance maternal and child health service utilization among pregnant women from rural Nepal: study protocol for a cluster randomised controlled trial
Source: BMC Health Serv Res. 2018 Jun 14;18:447. doi: 10.1186/s12913-018-3223-6 (PMC6001039; doi:10.1186/s12913-018-3223-6)
Supplement: Supplementary file 6 — Participant Consent Form. (DOCX 15 kb) [file 12913_2018_3223_MOESM6_ESM.docx]

**LETTER OF INTRODUCTION & CONSENT:**

**LETTER OF INTRODUCTION**

Hello. My name is ***Jitendra Kumar Singh***, and I am a student at Department of Community Medicine, Banaras Hindu University, India. I am conducting a study on “***Effect of Capacity building and technological intervention on utilization of MCH services and dietary intake among pregnant women in rural communities of Dhanusha district of Nepal***”. I will ask you questions about your health, household and dietary pattern. My assistant will take your anthropometry as well as some sample of blood from your arm to check your haemoglobin status. The questionnaire usually takes 30-45 minutes. Whatever information you shall provide will be kept strictly confidential and will not be shown to any other persons.

Participation in the study is voluntary and you can choose not to answer any individual question or all of the questions.

**Researcher:** Jitendra Kumar Singh

**Address:** Department of Community medicine, Institute of Medical Sciences,

Banaras Hindu University, Varanasi, UP, India.

**Email:** jsingdj@gmail.com **Cell Phone:** 091-8576855452/ 00977-9841462885

**LETTER OF CONSENT:**

By signing below, I ............................................................a resident of....................Dhanusha, Nepal, agrees to take part in this research study. I hereby declare that:

- I have been informed about the research and I have understood the benefits and

the risks involved.

- I have the chance to ask questions and all my questions have been adequately answered.
- I understand that taking part in this study is voluntary and I have not been

pressured to take part in it.

- I may choose to leave the study at any time and that I will not be penalized or

prejudiced in any way.

- I have been assured of confidentiality on any information that will be given.

...........................................................

Signature /Thumb Print of respondent: Date: ……………......….

.............................................................

Signature of investigator Date: ............................

...........................................................

Signature /Thumb Print of witness: Date: ……………......…......
